# PK/PD investigation of antiviral host matriptase/TMPRSS2 inhibitors in cell models

**DOI:** 10.1038/s41598-024-67633-2

**Published:** 2024-07-18

**Authors:** Dávid Gamba, Nicholas van Eijk, Katalin Lányi, Katalin Monostory, Torsten Steinmetzer, András Marosi, Anita Rácz, Dávid Bajusz, Diana Kruhl, Eva Böttcher-Friebertshäuser, Erzsébet Pászti-Gere

**Affiliations:** 1 Department of Pharmacology and Toxicology, University of Veterinary Medicine, István Utca 2, 1078 Budapest, Hungary; 2 Department of Food Hygiene, University of Veterinary Medicine, István Utca 2, 1078 Budapest, Hungary; 3 Institute of Enzymology, Research Centre for Natural Sciences, Magyar Tudósok 2, 1117 Budapest, Hungary; 4 Faculty of Pharmacy, Institute of Pharmaceutical Chemistry, Philipps University Marburg, Marbacher Weg 6, 35032 Marburg, Germany; 5 Virology Research Group, Department of Microbiology and Infectious Diseases, University of Veterinary Medicine, Hungária krt 23, 1143 Budapest, Hungary; 6 Institute of Materials and Environmental Chemistry, Research Centre for Natural Sciences, Magyar Tudósok 2, 1117 Budapest, Hungary; 7 Institute of Organic Chemistry, Research Centre for Natural Sciences, Magyar Tudósok 2, 1117 Budapest, Hungary; 8 Institute of Virology, Philipps-University Marburg, Hans-Meerwein-Str. 2, 35043 Marburg, Germany

**Keywords:** Proteases, Pharmacodynamics, Pharmacokinetics, Antiviral agents

## Abstract

Certain corona- and influenza viruses utilize type II transmembrane serine proteases for cell entry, making these enzymes potential drug targets for the treatment of viral respiratory infections. In this study, the cytotoxicity and inhibitory effects of seven matriptase/TMPRSS2 inhibitors (MI-21, MI-463, MI-472, MI-485, MI-1900, MI-1903, and MI-1904) on cytochrome P450 enzymes were evaluated using fluorometric assays. Additionally, their antiviral activity against influenza A virus subtypes H1N1 and H9N2 was assessed. The metabolic depletion rates of these inhibitors in human primary hepatocytes were determined over a 120-min period by LC–MS/MS, and PK parameters were calculated. The tested compounds, with the exception of MI-21, displayed potent inhibition of CYP3A4, while all compounds lacked inhibitory effects on CYP1A2, CYP2C9, CYP2C19, and CYP2D6. The differences between the CYP3A4 activity within the series were rationalized by ligand docking. Elucidation of PK parameters showed that inhibitors MI-463, MI-472, MI-485, MI-1900 and MI-1904 were more stable compounds than MI-21 and MI-1903. Anti-H1N1 properties of inhibitors MI-463 and MI-1900 and anti-H9N2 effects of MI-463 were shown at 20 and 50 µM after 24 h incubation with the inhibitors, suggesting that these inhibitors can be applied to block entry of these viruses by suppressing host matriptase/TMPRSS2-mediated cleavage.

## Introduction

During the recent decades, there is increasing emergence of highly contagious respiratory infections worldwide, driven mainly by the high connectivity of globalization that provides more opportunity for transmission and rapid spread of pathogens. Potential co-occurrence of multiple airborne viruses in human communities pose even more challenges for public health systems, accounting for higher mortality and abundant economic burden. Recent example is the simultaneous spread of the pandemic coronavirus SARS-CoV-2, seasonal influenza A viruses (IAV) and respiratory syncytial virus, that highlights the demand on strengthening preparedness and resilience to prevent airborne pathogens-caused pandemics.

In 2009, (H1N1)pdm09 influenza virus emerged and the World Health Organization (WHO) declared a pandemic in the same year due to the worldwide circulation causing more than 60 million cases with approximately 270,000 hospitalizations and 100,000–400,000 excess death within a year^1^. Other IAVs are also capable of causing severe infections with high morbidity and occasionally high mortality rates. Moreover, their broad host range and adaptability via reassortment as well as frequent mutations makes them particularly suited to cause zoonotic outbreaks and “spillover” into human populations, as demonstrated by certain H3N8, H5N1, H5N6 and H9N2 strains^2^.

The surface of the IAV envelope carries the spike-shaped glycoprotein hemagglutinin (HA), which is responsible for the binding of the virus to cell surface receptors and mediates uncoating, leading to the liberation of the viral genome into the cytoplasm through membrane fusion. An additional surface protein is neuraminidase (NA), which contributes to virus spread by cleavage of sialic acids from mucins in the respiratory tract^3–5^. Thereby, NA amplifies HA hemagglutination activity by cleavage of the oligosaccharides surrounding the receptor-binding site of HA^4^. Furthermore, it enables the release of progeny viruses from the cell surface after budding.

The fusion of the viral lipid envelope with the cell membrane can start only after the HA0 precursor is cleaved by host cell proteases into HA1 and HA2 subunits. TMPRSS2 has been identified as the major host cell protease which cleaves HA of mammalian IAVs, including human viruses of subtypes H1 and H3 in vitro^6,7^. Nonetheless, variations in the reliance on transmembrane protease serine 2 (TMPRSS2)-mediated activation have been observed when utilizing the same virus strain in cells derived from distinct species. For instance, H3N2 exhibited the capacity for multicycle replication in type II alveolar epithelial cells (AECII) from both TMPRSS2-deficient mice and their wild-type counterparts. Conversely, the suppression of TMPRSS2 activity in primary human AECII impeded the propagation of H3N2^8^. Other members of the type II transmembrane serine proteases (TTSP) family have also been identified as activators of the HA of specific IAVs. In the case of H9N2, it has been demonstrated that, in addition to TMPRSS2, human airway trypsin-like protease (HAT) and matriptase can activate the HA of this virus, indicating that inhibition of viral entry by H9N2 can be achieved through inhibition of each of the aforementioned TTSPs^9^.

In recent decades, another group of TMPRSS2-activated viruses, three members of the Coronaviridae family have emerged, each capable of inducing severe infections in humans. These include the severe acute respiratory syndrome coronavirus (SARS-CoV), which caused an outbreak in 2002/2003, the Middle East respiratory syndrome virus (MERS-CoV) in 2012, and the severe acute respiratory syndrome coronavirus 2 (SARS-CoV-2) that provoked COVID-19 pandemic^10^. It was also established that IAVs and coronaviruses are similar in their ability to infect human airways and their co-circulation can facilitate airborne pathogen-caused pandemic^11^.

Coronaviruses rely on the structural (S) protein to facilitate infection of receptor-bearing cells by mediating entry into host cells. This pivotal step in their life-cycle also significantly contributes to their pathogenicity^12,13^. The coronavirus surface spike S protein interacts with angiotensin-converting enzyme 2 (ACE2) through a two-step proteolytic cleavage process. The first cleavage occurs at the S1/S2 site, facilitated by host cell furin, while the second cleavage occurs at the S2’ site, within the S2 subunit. This second cleavage can be catalyzed by TMPRSS2 on the cell surface, or by cathepsin L within the endosomal compartment^13,14^. These enzymes, which catalyze these critical reactions, represent intriguing targets for the development of novel treatments. Combination of TMPRSS2 and furin inhibitors is also of key importance, as TMPRSS2 and furin cleave different sites of the S protein, yielding synergism in blocking SARS-CoV-2 cell entry^15,16^.

Various 3-amidinophenylalanine (Phe(3-Am))-based matriptase/TMPRSS2 inhibitors with potential antiviral effects against the aforementioned airborne pathogens have been evaluated in different cell models so far^17^. The investigated compounds did not influence redox homeostasis significantly. Enzyme kinetic studies revealed that less selective compounds such as MI-477 showed stronger affinity not only towards matriptase/TMPRSS2, which is essential for cleavage of influenza and corona viral glycoprotein, but towards coagulation factors such as thrombin predicting potential risk of bleeding. Plasma protein binding assays excluded the interaction between human serum albumin and studied Phe(3-Am) derivatives, and complex formation was only found with α-1 acidic glycoprotein in case of MI-472 and MI-477^17^.

The cytochrome P450 (CYP) 1, 2, and 3 isoenzymes play an essential role in xenometabolism due to their involvement in the biotransformation of approximately 80% of clinical drugs^18^. Understanding drug interactions with CYP isoenzymes provides information on mechanism of action, safety profile, and bioavailability^19,20^. In hepatocyte studies, CYP3A4 was shown to be inhibited by inflammatory cytokines such as interleukins- 1 and -6 (IL-1, IL-6) and tumor necrosis factor (TNF)-α, which are present in elevated concentrations during infections, including SARS-CoV-2 infections^18,21,22^. Some drugs used as antivirals in SARS-CoV-2 infections, like remdesivir and Paxlovid (ritonavir-boosted nirmatrelvir), have inhibitory effects on CYP3A4, as well as on CYP1A2, CYP2C9, CYP2C19, and CYP2D6^18^. Thus, there is potential for drug-induced hepatotoxicity during SARS-CoV-2 infections due to virus-induced inflammation and the concomitant usage of antiviral drugs for treatment of the infection^21^. To evaluate the effects of pharmaceuticals on CYP isoenzymes, primary hepatocytes, hepatic microsomes, and recombinant human CYP isoenzymes are used extensively to reveal CYP interactions with tested pharmacons, this being the most common cause of drug-drug interactions^23^.

Currently there is no predicted pharmacokinetic (PK) and CYP-interaction data available for Phe(3-Am)-based matriptase/TMPRSS2 inhibitors, such as MI-21, MI-472, MI-485, MI-1903, and MI-1904, in human hepatic models. In this study, the rates of CYP3A4 modulation of these compounds were determined in human liver microsomes via fluorometric determination of % enzyme activity loss. Over the chosen time window (0–120 min), the pharmacokinetic behavior of the inhibitors in human primary hepatocytes was also evaluated using liquid chromatography-tandem mass spectrometry (LC–MS/MS) measurements to assess the effects of certain chemical structures on biotransformation rates of these Phe(3-Am)-derived inhibitors. Additionally, in vitro intrinsic clearance (Cl_int_) values were determined for each MI compound, and in vivo PK parameters, such as predicted Cl (Cl_H_), hepatic extraction ratio (E_H_), and bioavailability (F) were predicted. Antiviral capacity of certain Phe(3-Am)-based matriptase/TMPRSS2 inhibitors against IAV subtypes H1N1 and H9N2 was also determined using an in vitro post-infection antiviral plaque assay.

## Methods

### Effects of the inhibitors on the activity of various human microsomal CYP isoenzymes

The BioVision CYP assays (BioVision, Inc., Kampenhout, Belgium) contain non-fluorescent substrates selective for CYP1A2, CYP2C9, CYP2C19, CYP2D6, or CYP3A4, and the highly fluorescent metabolites formed by CYP enzymes are detectable by a fluorometer. The CYP-selective inhibitors, α-naphthoflavone (α-NF; CYP1A2, 6 µM), tienilic acid (CYP2C9, 60 µM), ( +)-N-3-benzylnirvanol (CYP2C19, 30 µM), ketoconazole (KCZ, CYP3A4, 30 µM), and quinidine (CYP2D6, 3 µM) were used as positive controls in these experiments.

Protein content of human hepatic microsomes (Gibco, Biocenter, Szeged, Hungary) was determined by the bicinchoninic acid protein assay kit (Pierce BCA kit, Thermo Fisher Scientific, Waltham, MA, US) and a 100 µg/well protein was used in each assay. A suspension of human hepatic microsomes (2500 µL) contained 30 µL human hepatic microsomes (20 mg/mL) and nicotinamide adenine dinucleotide phosphate (NADPH)-generating system (50 µL) in assay buffer. The microsomal suspension aliquots (50 µL) were combined with either the reference inhibitors selective for CYP enzymes (α-naphthoflavone, 30 µM; tienilic acid, 300 µM; ( +)- N-3-benzylnirvanol, 150 µM; ketoconazole, 150 µM; quinidine, 15 µM; each 20 µL) or the protease inhibitors (MI-21, MI-463, MI-472, MI-485, MI-1903, and MI-1904, each 20 µL) at the final concentration of 250 µM except for CYP3A4 where the final concentrations were 50, 125, and 250 µM. The inhibitor-free buffer (20 µL) was added as a control experiment. The background control contained 70 µL of assay buffer without microsomes and protease inhibitors. The reaction was started by adding the mixture of assay buffer and NADPH-generating system to microsomes + inhibitors. The mixture of the suitable CYP substrate/NADPH-generating system (30 µL) was added to each well after incubation for 15 min at 37 °C, yielding 100 µL as final reaction volume.

A fluorometer (Victor X2 2030, Perkin Elmer, Waltham, MA, US) using wavelengths of λ_ex/em_ = 406/468 nm for CYP1A2 and for CYP2C19, λ_ex/em_ = 415/502 nm for CYP2C9, λ_ex/em_ = 390/468 nm for CYP2D6, and λ_ex/em_ = 535/587 nm for CYP3A4 assays was applied to measure the fluorescence intensities.

### Ligand docking

For docking the small molecules into the active site and peripheral pocket of CYP 3A4, the PDB structures 6MA8^24^ and 1W0F^25^ were used, respectively. The ligands were prepared with Schrödinger Ligprep: briefly, protomers are generated in a pH range of 7.4 ± 1.5, and stereoisomers are enumerated for each ligand^26^. The ligands were then docked with Glide SP into the unique CYP 3A4 structures^27,28^.

### Preparation of human primary hepatocyte suspensions

Cryopreserved human primary hepatocytes were acquired from Primacyt (Primacyt, Schwerin, Germany). Using cryopreserved hepatocyte-thawing medium (Primacyt, Schwerin, Germany), hepatocytes were thawed, and cells were then centrifuged (10 min, 100×*g*, 20 °C) for the removal of cell debris in supernatants. Hepatocyte viability was examined by the Trypan blue exclusion method, which confirmed that over 90% of the cells were alive. Following cell counting in a Bürker chamber to the appropriate concentration (original concentration of the viable hepatocytes was 10^7^ cells/mL), cell suspensions were then diluted using serum-free maintenance medium (Primacyt, Schwerin, Germany).

### Incubation of human hepatocytes with inhibitors

**Figure 1:**
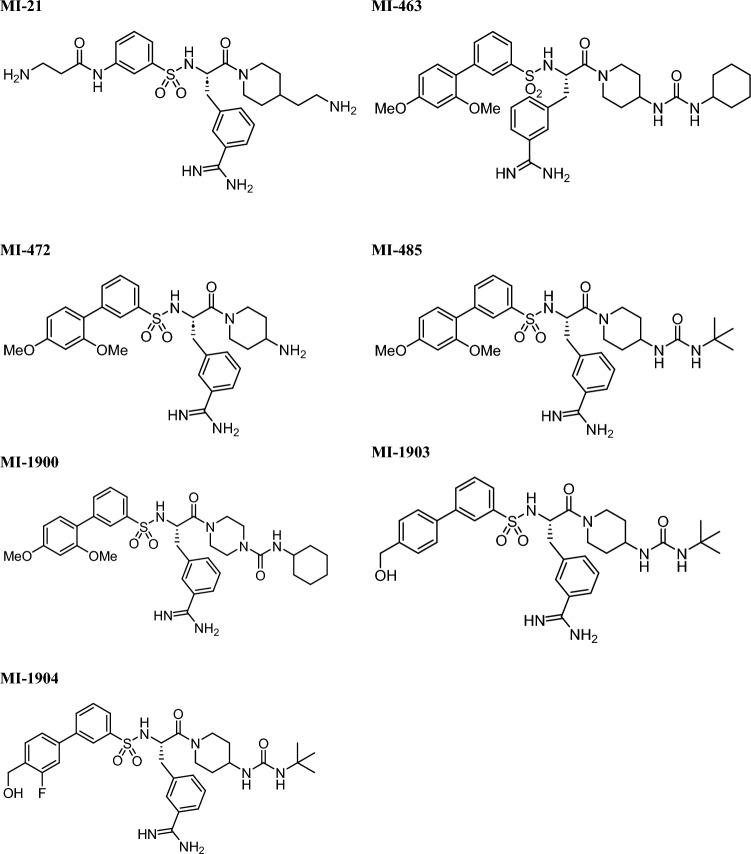
Chemical structures of the applied inhibitors.

Stock solutions of inhibitors MI-21, MI-463, MI-472, MI-485, MI-1900, MI-1903 and MI-1904 (10 mM) in DMSO were kept at − 20 °C. The structures of the protease inhibitors are presented in Fig. 1. After incubation with human hepatocytes, time courses of the unchanged amounts of inhibitors (in %) were measured. Each of the compounds was incubated in a cell suspension (at the concentration of 5 × 10^5^ cells/mL in 6-well plates) at constant and low-speed shaking at 37 °C in a humid atmosphere that contained 5% CO_2_. The 10 µM solutions of the inhibitors were freshly prepared with maintenance medium from the stock before each experiment. DMSO concentration did not exceed 0.1% v/v in the final solutions. Viability of human primary hepatocytes MI-21, MI-472, MI-485, MI-1903 and MI-1904 was previously evaluated at 50 µM for 24 h^17^. In this study, the lack of cytotoxicity was confirmed in primary human liver cells exposed to MI-463, MI-1900 at 50 µM and DMSO at 0.5% v/v as solvent control for 24 h (Fig. S1).

Cells were incubated using serum-free maintenance medium either with or without each selected inhibitor for 120 min. At several time points (0, 15, 30, 60, 120 min), each incubation mixture was sampled (aliquots: 0.15 mL) and terminated by the adding of 0.1 mL of ice-cold acetonitrile. By centrifugation, cell debris was separated, and the supernatants were analyzed for quantitation of the remaining amount of the inhibitors by LC–MS/MS.

### Quantification of unchanged inhibitors

At various time points, an aliquot of the incubation mixture (150 µL) was taken for the analysis of the parent compounds MI-463, MI-472, MI-485, MI-1900, MI-1903, and MI-1904, and 330 µL chilled acetonitrile containing triphenylphosphate (TPP; 12,000 ng/mL as internal standard for the quantitation) was added. For compound MI-21, an aliquot of 120 µL from the mixture was removed for analysis after the required incubation time. 330 µl chilled acetonitrile was used. The samples were vortex-mixed and centrifuged at 13.300 rpm and at 4 °C for 10 min. The supernatant was filtered through a PVDF syringe filter of 0.22 m pore diameter into a chromatographic vial and analysed by LC–MS/MS. A Shimadzu LCMS 8030 + system was used for the analysis and was operated with an electrospray ion source (ESI) in positive multiple reaction monitoring (MRM) mode. The MS interface voltage was 4.5 kV; interface temperature 350 °C; desolvation line 300 °C; heat block 400 °C (for MI-21 350 °C); detector 1.78 kV, nebulizing gas (N_2_) 3 L/min, drying gas (N_2_) 15 L/min (for MI-21 drying gas (N_2_) was 10 L/min); collision gas (Ar) 230 kPa. A Phenomenex Kinetex C18 EVO 100 × 4.6 mm (2.6 µm particle size) column (Torrance, USA) was used for the chromatographic separation except in case of MI-21 where Phenomenex Kinetex C18 EVO 50 × 4.6 mm column was used. Gradient elution was used with eluents A: 50 mM ammonium acetate in water (pH 5 set by acetic acid) and B: 0.1 v/v% formic acid in acetonitrile. The flow rate was set to 0.4 mL/min, the injected volume was 10 µL. The column was thermostated to 30 °C, and the autoinjector to 6 °C. For the determination of MI-21, gradient elution was applied with eluents A: 0.1 v/v% formic acid in water and B: 0.1 v/v% formic acid in acetonitrile. The flow rate was set to 0.5 mL/min, the injected volume was 10 µL. The column was thermostated to 40 °C, and the autoinjector to 6 °C.

### Assessment of pharmacokinetic parameters

The intrinsic clearance for hepatocytes (Cl_int_) [ml min 5 × 10^5^cells) ^-1^] was determined from the decrease in the concentration of inhibitors^29^. In vivo PK parameters (predicted clearance Cl_H_, hepatic extraction ratio E_H_, bioavailability F) were calculated according to the previously published procedure^30^. To predict Cl_int per whole liver (g)/bw (kg)_, the cell concentration in the liver, the average liver weight, and average body weight parameters, and to calculate Cl_H_, the hepatic flow rate, plasma/blood ratio (Table 1) and fu = 1 were used^31,32^.

**Table 1:** Physiological parameters for prediction of human clearance values.

Parameter	Human
Number of hepatocytes in liver (× 10^6^ cells/g liver)	139
Liver weight (g)	1660
Body weight (kg)	70
Liver blood flow (mL/min/kg)	20.7
Plasma/blood ratio	0.57

### Cells for propagation of viruses

Growth and incubation of cell lines used in the experiments were carried out in a humidified incubator at 37 °C and 5% CO_2_. Madin-Darby canine kidney cells II (MDCK-II) were maintained in Dulbecco’s modified Eagle medium (DMEM; Gibco) supplemented with 10% fetal calf serum (FCS), 5% glutamine and 5% penicillin/streptomycin. Calu-3 human airway epithelial cells (ATCC number HTB55) were cultured in DMEM/F-12 Ham (1:1) (Gibco) with 10% FCS, glutamine, and penicillin/streptomycin, with fresh culture medium replenished every 2–3 days. H9N2 IAV strain A/quail/Shantou/782/00 and A/Hamburg/5/09 (H1N1pdm) were propagated in MDCK-II cells using infection medium (DMEM supplemented with 0.1% bovine serum albumin (BSA) (Sigma-Aldrich), 5% glutamine and 5% penicillin/ streptomycin) containing 1 μg/mL tosyl phenylalanyl chloromethyl ketone (TPCK)-treated trypsin (Sigma-Aldrich). Cell supernatants were cleared from cell debris by low-speed centrifugation and stored at − 80 °C.

### Antiviral assay and viral multicycle replication analysis

MDCK-II cells were seeded in 24-well plates and grown to > 95% confluence. Followed by gentle washing with PBS, the cells were infected with H9N2 at a multiplicity of infection (MOI) of 0.01 for 1 h at 37 °C. To treat the cells with inhibitors, the inoculum was removed, and cells were washed again with PBS. Cell cultures were then covered with fresh infection medium that did or did not (control) contain protease inhibitors (at final concentrations of 20 and 50 µM), or as vehicle control, DMSO (≤ 5% v/v), and incubated at 37 °C and 5% CO_2_. For analysis of virus spread at 24 h post infection (p.i.), the cells were fixed with paraformaldehyde and immunostained against the viral nucleoprotein (NP) using a monoclonal mouse anti-NP antibody (Abcam), peroxidase-conjugated secondary antibodies and TrueBlue Peroxidase substrate (KPL) as described in previous publications^33,34^. Visualization of immunostained cells was achieved using the ChemiDoc XRS + system and the Image Lab software (Version 6.1 for Windows PC, https://www.bio-rad.com/de-de/product/image-lab-software?ID=KRE6P5E8Z). For analysis of virus replication kinetics, samples were taken from the supernatant and stored at − 20 °C at 24 and 48 h p.i., then virus titration was performed using a plaque assay on MDCK-II cells with Avicel overlay as described previously^8,35^. For studies of the H1N1pdm virus multicycle replication kinetics, Calu-3 cells were seeded in 24-well plates and grown to near-confluence. Cells were inoculated with H1N1 at a MOI of 0.001 for 1 h, then washed with PBS and incubated in infection medium with or without protease inhibitors at 20 and 50 µM or DMSO for 48 h. At 24 and 48 h p.i., supernatants were collected, and viral titers were determined by plaque assay in MDCK-II cells.

### Cell viability assay

Cell viability was assessed by measuring the cellular ATP content using the CellTiterGlo® luminescent cell viability assay (Promega). MDCK-II cells grown in 96-well plates were incubated with 50 μM of the indicated inhibitor in triplicates for 24 h. Untreated and DMSO-treated cells were used as controls. Subsequently, cells were incubated with the substrate according to the manufacturer’s protocol and luminescence was measured using a 96-well plate (Nunc). The absorbance values of inhibitor-treated cells were converted to percentages by comparison to untreated control cells, which were set at 100% cell viability.

### Statistical analysis

Data of normal distribution and homogeneity of variances were confirmed prior to the application of one-way ANOVA and Tukey’s post hoc test using the R version 4.0.4 software package (Vienna, Austria, 2021, https://cran.r-project.org/bin/windows/base/old/4.0.4/). The results were expressed as means ± SEM, and differences between results were evaluated to be significant if p was < 0.05 (*p < 0.05, **p < 0.01, ***p < 0.001). Every measurement was repeated at least in triplicate.

## Results

### Effects of the inhibitors on the activity of various human microsomal CYP isoenzymes

**Figure 2:**
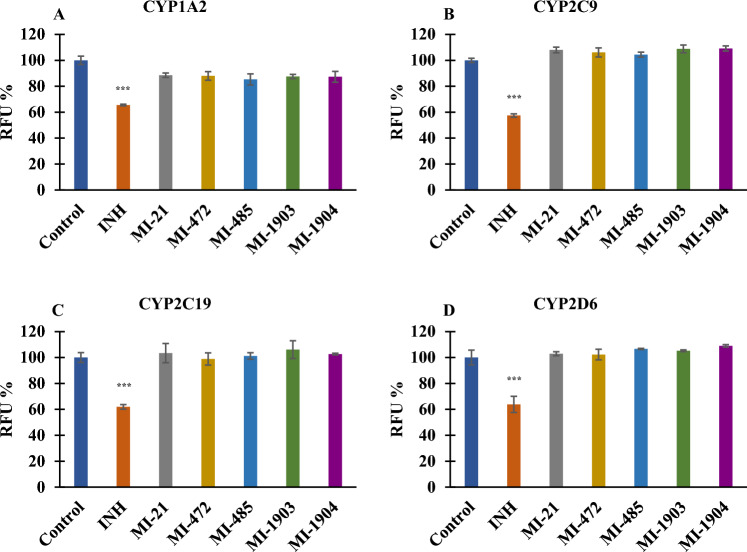
Inhibition of CYP activities [CYP1A2 (**A**), CYP2C9 (**B**), CYP2C19 (**C**), and CYP2D6 (**D**)] by protease inhibitors. The reference inhibitors (INH) α-naphthoflavone (CYP1A2, 6 µM), tienilic acid (CYP2C9, 60 µM), (+)-N-3-benzylnirvanol (CYP2C19, 30 µM), and quinidine (CYP2D6, 3 µM) significantly suppressed CYP enzyme activities (***p < 0.001). No significant inhibition was found on investigated CYP isoenzymes exposed to matriptase/TMPRSS2 inhibitors (p > 0.05). Relative fluorescence units (RFUs) of the metabolites formed in CYP enzyme reactions (λ_ex/em_ = 406/468 nm for CYP1A2 and for CYP2C19, λ_ex/em_ = 415/502 nm for CYP2C9, and λ_ex/em_ = 390/468 nm for CYP2D6) are depicted as mean values ± S.E.M. (n = 3).

The effect of the inhibitors on the activities of human CYP enzymes responsible for drug metabolism (CYP1A2, CYP2C9, CYP2C19, CYP2D6 and CYP3A4) was examined in liver microsomes. The reference inhibitors (α-naphthoflavone at 30 µM; tienilic acid at 300 µM; (+)- N-3-benzylnirvanol at 150 µM; ketoconazole at 150 µM, and quinidine at 15 µM) demonstrated a significant decrease in the respective CYP enzyme activity following a 15 min incubation at 37 °C (***p < 0.001), whereas the activities of CYP1A2, CYP2C9, CYP2C19, and CYP2D6 enzymes were not inhibited by any of the tested protease inhibitors at 50 µM (p > 0.05) (Fig. 2).

**Figure 3:**
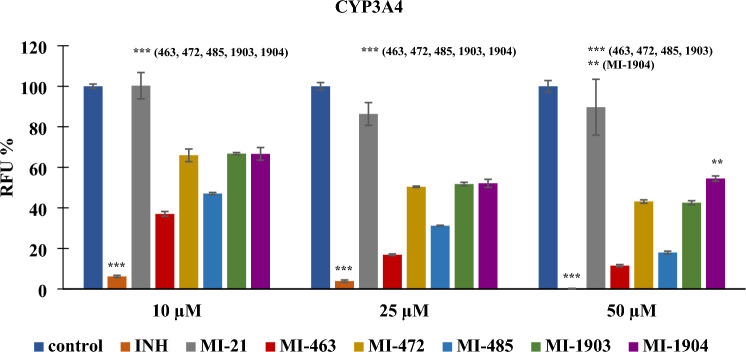
Effect of protease inhibitors at various concentrations (10, 25, and 50 µM) on CYP3A4 enzyme activity. The reference inhibitor (INH) ketoconazole (30 µM) significantly suppressed the activity of CYP3A4 (***p < 0.01). MI-463 (***p < 0.01), MI-472 (***p < 0.01), MI-485 (***p < 0.01), MI-1903 (***p < 0.01), and MI-1904 (10 and 25 µM, ***p < 0.01; 50 µM, **p < 0.01) all had a significant inhibitory effect on CYP3A4. MI-21 at 10, 25 and 50 µM did not significantly suppress CYP3A4 activity (p > 0.05). Relative fluorescence units (RFUs) of each compound (λ_ex_/_em_ = 535/587 nm) are depicted as mean values ± S.E.M. (n = 3; MI-21, n = 4).

In contrast, at all three concentrations (10, 25, and 50 µM) the inhibitors provoked a significant decrease in CYP3A4 activity (***p < 0.001; 50 µM MI-1904, **p < 0.01), with the exception of MI-21 as it did not cause significant reduction in CYP3A4 function at 10 µM, 25 µM and 50 µM concentrations (p > 0.05). However, MI-463 and MI-485 seemed to act as potent inhibitors even at the lowest applied concentration (Fig. 3).

### Interaction of matriptase inhibitors with CYP3A4 by ligand docking

**Figure 4:**
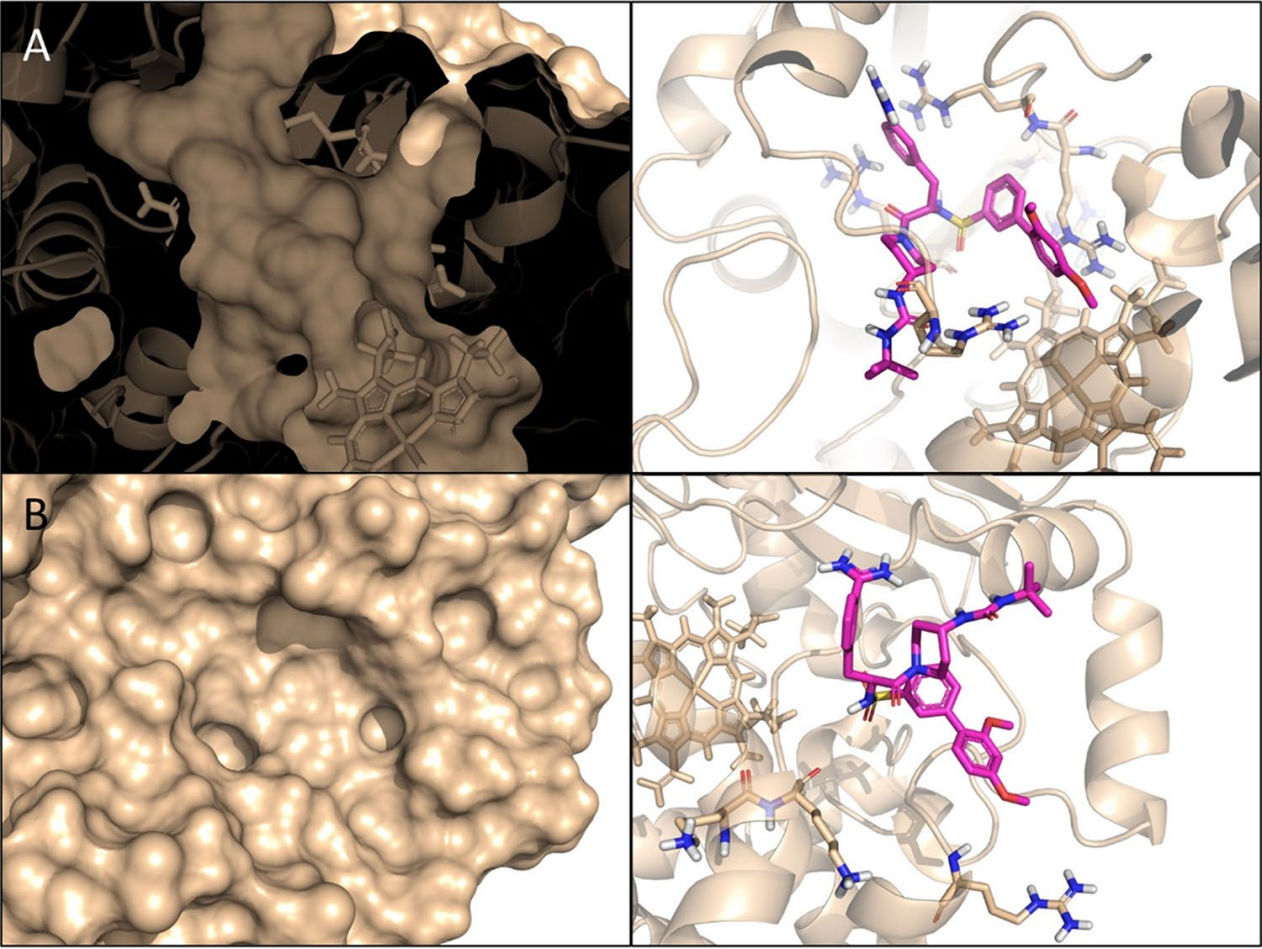
(**A**) The active site (left: cross-section of surface view, right: predicted binding mode of MI-485) is more buried and is lined with a cluster of positively charged arginine sidechains (highlighted as sticks) that should provide a stronger repulsive force against the most heavily charged molecule MI-21. (**B**) The solvent-exposed peripheral pocket (left: surface view, right: predicted binding mode of MI-485) is in general more difficult to target with small molecules, and does not exhibit any structural feature that would clearly explain the activity difference between MI-21 and the rest of the series. (Predicted binding poses in the two sites for the rest of the series of inhibitors are included in Supplementary Fig. S2).

To understand the influence of the tested protease inhibitors on CYP 3A4 activity, and specifically, the lack of inhibition by compound MI-21, we have analyzed a set of basic molecular properties of the compounds, as well as their binding modes in the active site and the peripheral pocket of CYP3A4, predicted by ligand docking. To note, all compounds of the series contain one or two positively charged groups like ammonium, amidine, or guanidine, except for inhibitor MI-21, which possesses three strongly basic groups. Inspecting the two possible binding sites at CYP3A4, we noticed that the active site is more compact and buried than the peripheral pocket, and it is lined with a tight cluster of five positively charged arginine residues (Fig. 4A). The proximity of this cluster should provide a stronger repulsive force against the most heavily charged MI-21 than the other members of the series, resulting in a weaker affinity to this binding site. (In comparison, compound MI-485, shown in Fig. 4, has only one positively charged group.) By contrast, the peripheral pocket is a surface site with a much more diffuse distribution of charged sidechains, which should be able to accommodate all compounds to the same extent (Fig. 4B). Additionally, shallow sites are harder to target with small molecules. Based on these observations, we propose that this series binds primarily to the active site and exerts its inhibitory activity in a direct, orthosteric manner.

### Depletion of protease inhibitors

**Figure 5:**
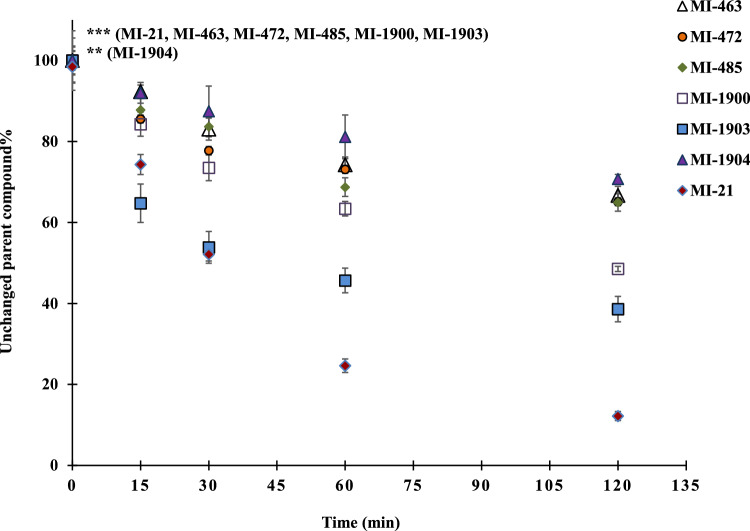
Time course of depletion of the protease inhibitors in primary human hepatocytes. Relative percentage values of the unchanged amount of parent compounds were calculated by dividing the concentrations of the inhibitor measured at the given time point by the mean starting amount of the inhibitors in the treatment solution (mean ± S.E.M.); n = 3/group. Asterisks indicate significant differences compared to starting values of the indicated inhibitors at 120 min in parentheses (**p < 0.01, ***p < 0.001).

The tested inhibitors demonstrated substantial depletion as early as the 30-min sampling interval (p_MI-21, MI-463, MI-472, MI-485, MI-1900, MI-1903, 30 min_ < 0.001 (Fig. 5) using suspension of primary human hepatocytes. It should be noted that inhibitor MI-1904 displayed the lowest rate of elimination, and significant depletion was observed only after the 120-min sampling interval (p_MI-1904, 120 min_ = 0.00406).

Significant biotransformation of inhibitor MI-21 was observed with marked loss of the parent compounds compared to all other inhibitors except MI-1903 at the 30-min sampling interval (p_MI-463, MI-472, MI-485, MI-1900, MI-1904, 30 min_ < 0.001). At the 60-min and 120-min sampling points, however, it was significantly more depleted than all other inhibitors (p_MI-463, MI-472, MI-485, MI-1900, MI-1903, MI-1904, 60 and 120 min_ < 0.001). Inhibitor MI-1903 also exhibited significant depletion rates compared to the other inhibitors at the 30-min (p_MI-463, MI-472, MI-485, 30 min_ < 0.001, p_MI-1900, 30 min_ = 0.0050, p_MI-1904, 30 min_ < 0.001), 60-min (p_MI-463, MI-472, MI-485, 60 min_ < 0.001, p_MI-1900, 60 min_ = 0.00251, p_MI-1904_ < 0.001), and 120-min (p_MI-463, MI-472, MI-485, MI-1904, 120 min_ < 0.001,) sampling points. Similarly, inhibitor MI-1900 exhibited an extensive biotransformation, displaying significantly higher depletion at the 60-min sampling interval than compounds MI-463 and MI-1904 (p_MI-463, 60 min_ 0.03246, p_MI-1904, 60 min_ 0.00242), and at the 120-min sampling interval than compounds MI-463, MI-472, MI-485, and MI-1904 (p_MI-463, MI-472, MI-485, MI-1904, 120 min_ < 0.001).

Inhibitor MI-21, with its increased depletion rate compared to all other tested MI compounds (p_MI-463, MI-472, MI-485, MI-1900, MI-1903, MI-1904, 120 min_ < 0.001), retained only 16.6 ± 1.6% of the parent compounds at the 120-min sampling point. Inhibitors MI-1900 and MI-1903 were significantly faster depleted compared to the remaining MI compounds after the 120-min incubation (MI-1900, MI-1903: p_MI-463, MI-472, MI-485, MI-1904, 120 min_ < 0.001), retaining 48.6 ± 1.5% and 38.6 ± 5.5% of the 0-min concentrations of the parent compounds, respectively. Inhibitors MI-463, MI-472, MI-485, and MI-1904 retained the most substantial proportions of the initial concentrations (66.7 ± 5.5%, 65.1 ± 5.5%, 64.9 ± 5.3% and 70.8 ± 8.6%%, respectively).

A subtle difference in chemical structures between inhibitors MI-1903 and MI-1904 (with a fluoro substituent on biphenyl side chain in MI-1904) seems to have a significant impact on their respective depletion rates (p_120 min_ < 0.001). Inhibitor MI-1903 displayed a rapid depletion, whereas MI-1904 was the least depleted compound.

### Pharmacokinetic behavior of the inhibitors

Based on predicted in vivo pharmacokinetic parameters, all seven inhibitors exhibited intermediate extraction characteristics (Table 2). However, substantial variations of in vitro pharmacokinetic parameters (half-life and intrinsic clearance) emerged. Compounds MI-472 and MI-1904 displayed the longest half-lives at 230.4 and 239.4 min, respectively, while the half-lives of MI-21 (31.2 min) and MI-1903 (45.2 min) were notably shorter than that of the others. Consequently, the intrinsic clearance values also differed with compounds MI-21 and MI-1903 showing markedly higher intrinsic clearance at 146.56 ml/min/kg and 101.0261 ml/min/kg, respectively – more than twice of the Cl_int_ value for MI-1900, which had the next highest intrinsic clearance (42.34 ml/kg/min). While there were slight differences in extraction ratios and bioavailability values, compounds MI-21 and MI-1903 were outstanding, with the highest extraction ratios and the lowest bioavailability percentages. Compounds MI-463, MI-472, and MI-1904 exhibited the highest stability, characterized by notably prolonged half-lives, modest intrinsic clearance values, intermediate extraction ratios, and bioavailability.

**Table 2:** In vivo human PK parameters for the inhibitors.

Compound	Half-life, t_1/2_ (min)	CL_int_ (ml/min/kg)	Predicted Cl, CL_H_	Extraction ratio E_H_	Bioavailability, F (%)	Classification (extraction type)
MI-21	31.2	146.56	10.92	0.53	47.25	Intermediate
MI-463	214.2	21.34	7.60	0.37	63.30	Intermediate
MI-472	230.4	19.83	7.40	0.36	64.26	Intermediate
MI-485	171.8	26.60	8.17	0.39	60.51	Intermediate
MI-1900	107.9	42.34	9.23	0.45	55.42	Intermediate
MI-1903	45.2	101.03	10.56	0.51	48.96	Intermediate
MI-1904	239.4	19.09	7.29	0.35	64.77	Intermediate

### Inhibitory effect of protease inhibitors on matriptase-catalyzed activation of H9N2 influenza virus

**Figure 6:**
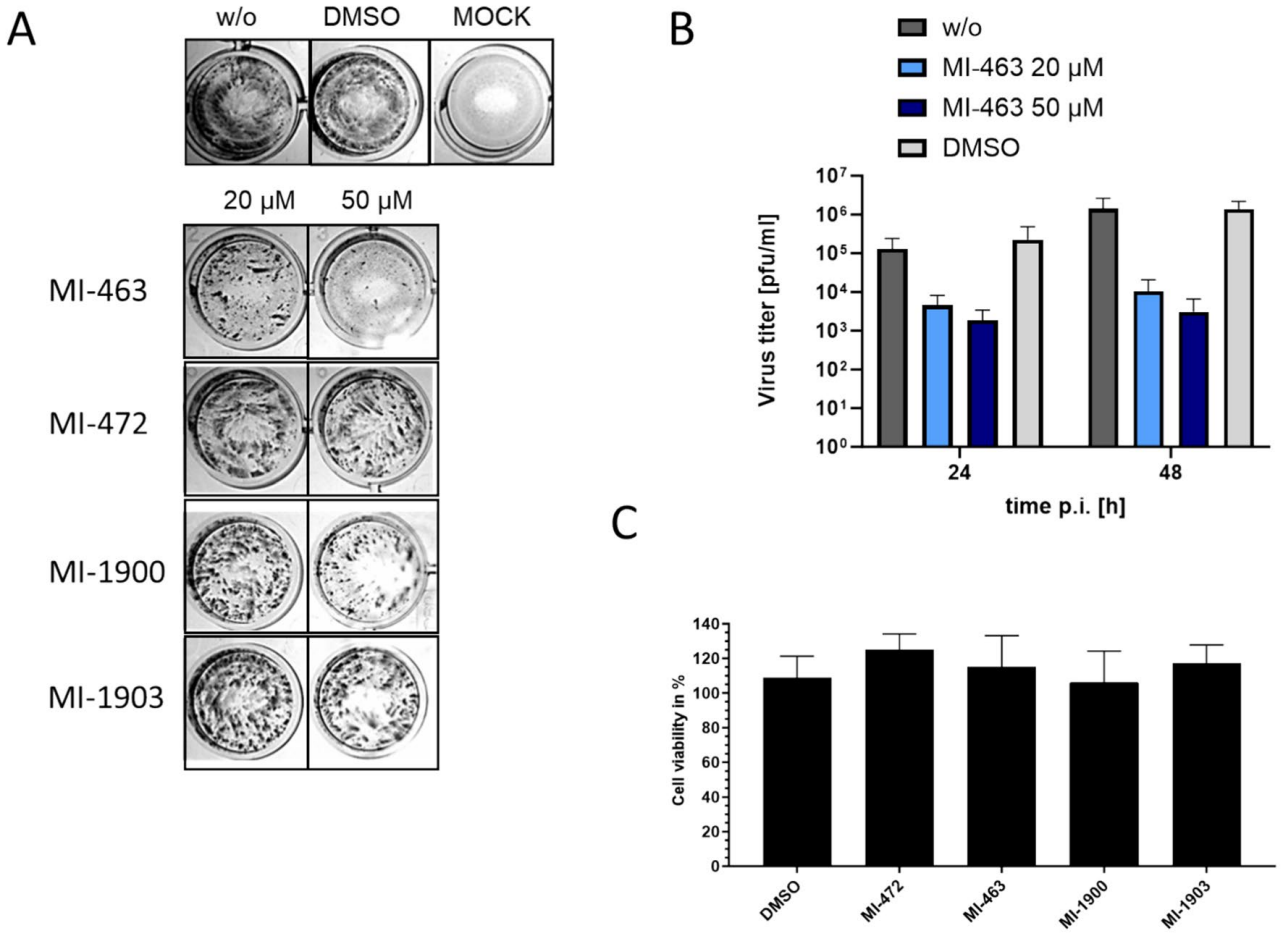
Inhibition of H9N2 multicycle replication in MDCK-II cells by matriptase inhibitors. (**A**) MDCK-II cells were infected with H9N2 virus at a MOI of 0.01 and incubated in the presence of the indicated inhibitors for 24 h. Untreated (w/o) and non-infected (MOCK) cells served as controls. Virus-infected cells were immunostained against the viral nucleoprotein at 24 h. (**B**) MDCK-II cells were infected with H9N2 IAV at a MOI of 0.01 and incubated in the presence of inhibitor MI-463 for 48 h. At indicated time points virus titers in supernatants were determined as plaque-forming units (pfu)/mL. Untreated (w/o) and DMSO-treated cells served as controls. Data are mean values + SD (n = 4). (**C**) Effect of inhibitor treatment on cell viability. MDCK-II cells were treated with the indicated protease inhibitor (50 μM) for 24 h. Untreated cells (w/o) and DMSO treated cells were used as controls. Cell viability of untreated cells was set as 100%. Results are mean values ± SD (n = 3).

H9N2 influenza strain A/quail/Shantou/782/00 has been shown to be activated by the TTSP matriptase in MDCK-II cells^9^. Thus, we tested the antiviral activity of protease inhibitors MI-463, MI-472, MI-1900, and MI-1903 in MDCK-II cells infected with IAV H9N2. Cells were infected by the virus at a MOI of 0.01 and then incubated in the presence and absence of inhibitors at 20, and 50 µM. Proteolytic activation and spread of the virus with and without inhibitor treatment was determined after 24 h by immunostaining the cells against the viral nucleoprotein (Fig. 6A).

The strongest antiviral efficacy among the inhibitors was displayed by MI-463, as it fully prevented formation of viral foci at 50 µM and already showed a marked reduction of viral spread at 20 µM. At 50 µM, MI-1900 also presented strong suppression of viral spread, while both MI-472 and MI-1903 only slightly reduced formation of viral foci.

For quantification of the antiviral efficacy of the inhibitor showing the greatest inhibition on H9N2 replication, MI-463, a plaque assay was used to determine the viral titers in the cell culture supernatants. After 24 h, viral titers measured in the MI-463-treated cell cultures were reduced by approximately 1.4 log (20 µM) or 1.8 log (50 µM) compared to untreated controls. The effect was enhanced after 48 h to a reduction of 2.1 and 2.6 logs by the two concentrations of the compounds, respectively (Fig. 6B). Cell viability was not affected by all inhibitors at a concentration of 50 µM (Fig. 6C).

### Impact of protease inhibitors on H1N1 influenza virus titers

**Figure 7:**
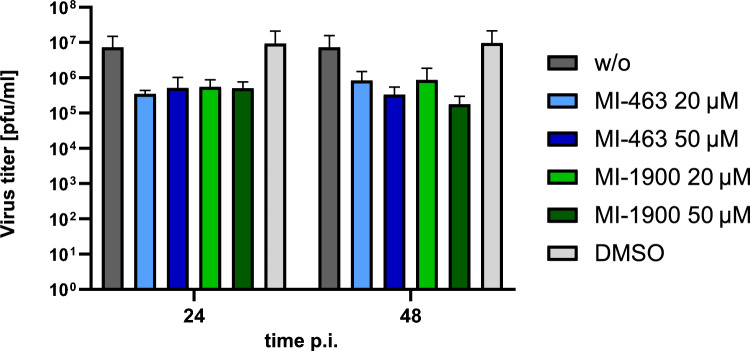
Inhibition of H1N1pdm multicycle replication in human airway cells by protease inhibitors. Calu-3 human airway cells were infected with H1N1pdm virus at a MOI of 0.001 and incubated in the presence of the indicated inhibitors for 48 h. Untreated (w/o) and DMSO-treated cells served as controls. Virus titers in supernatants were determined as plaque-forming units/mL (pfu/mL) at 24 and 48 h. Data are mean values ± SD (n = 3).

TMPRSS2 was identified as major activating protease of IAV in human airway cells^6,8^. In addition to MI-463, which could inhibit the activation of IAV by TMPRSS2, MI-1900 was also tested, since this inhibitor was also shown to reduce H9N2 virus replication in MDCK-II cells to some extent (Fig. 6A). Human Calu-3 airway cells were infected with the 2009 pandemic virus A/Hamburg/05/09 (H1N1pdm) at a low MOI and then incubated in the absence or presence of MI-463 or MI-1900 at the indicated concentrations for 48 h. At 24 and 48 h p.i., cell supernatants were collected, and virus titers were determined by plaque assay. Antiviral activity of MI-463 against H1N1, while still notable, was markedly lower than its efficacy against H9N2, with reduction of viral titers by 0.95–1.34 logs at different concentrations and incubation times. MI-1900 showed results comparable to those of MI-463, while reaching a slightly greater, 1.62 logs reduction of H1N1 titers at 50 µM after 48 h (Fig. 7).

## Discussion

To evaluate drug interaction potential of the tested TTSP inhibitors, their inhibitory effects on CYP activities were assessed in human liver microsomes. The present work was the first to evaluate the possible interactions between drug-metabolizing CYP isoenzymes and MI-21, MI-472, MI-485, MI-1903 or MI-1904. No significant CYP inhibitory potential of these inhibitors was found towards CYP1A2, CYP2C9, CYP2C19, and CYP2D6, similarly as observed with other Phe(3-Am) derivatives such as MI-463 and MI-1900^36^. Interestingly, another study showed significant inhibition of both monkey and rat CYP1A2 enzymes by inhibitors MI-463 and MI-1900, whereas no significant inhibition on beagle dog CYP1A2 was observed, indicating species-dependent inhibitory potential of the inhibitors on CYP isoenzymes^37^.

In the present study applying human microsomal enzymes, MI-463, MI-472, MI-485, MI-1903, and MI-1904 all showed significant inhibition on CYP3A4 activity, whereas MI-21 was the only compound tested that had no significant impact on this isoenzyme. It has been previously demonstrated that compound MI-1900 also exhibited substantial inhibitory effects only on CYP3A4, as determined through fluorometric assays, and effectively suppressed the isoenzyme activity^36^. Furthermore, MI-463 and MI-485 were found to be one of the most potent inhibitors of CYP3A4 and displayed a concentration-dependent inhibition of the enzyme. This may be related to the presence of both an N-terminal dimethoxy-substituted biphenyl-3-sulfonyl group and a C-terminal cyclohexyl-ureido moiety in MI-463 and a C-terminal tert-butyl ureido moiety in MI-485. The somewhat lower inhibitory potential of MI-1904 towards CYP3A4 may be attributed to the presence of a fluorine substituent, which was the only difference between MI-1904 and MI-1903.

The unique structural feature of inhibitor MI-21 containing three basic groups should result in a strong repulsive force by the arginine residues present in the active site of CYP3A4. Based on these structural differences between compound MI-21 and the rest of the series, as well as the two possible binding sites, we propose that this series binds primarily to the active site and exerts its inhibitory activity in a direct, orthosteric manner. We should note that a definitive conclusion on this matter would require further experimental work, while our suggestion is based solely on a visual inspection of the binding sites in the available X-ray structures, as well as the binding modes generated by computational modeling.

It was previously proven that inhibitor MI-21 appeared to be a potential drug candidate possessing no cell toxicity at a concentration of 50 µM^17^. In contrast, it displayed the fastest rate of depletion in comparison to the other inhibitors in this study using human hepatocytes. This might be due to the structural differences mentioned above that set it apart from the other inhibitors. Inhibitors MI-1900 and MI-1903 also exhibited a significantly accelerated rate of depletion compared to the other tested compounds. While this finding might initially imply a potential link to the C-terminal ureido group, this trait did not translate to the lower depletion rate found for inhibitor MI-1904, which features the same C-terminal ureido group. Consequently, it is reasonable to deduce that other structural features among these compounds must play a pivotal role in governing their respective rates of depletion.

The sole disparity within the molecular composition of inhibitors MI-1903 and MI-1904 is the introduction of a fluorine substituent in the latter compound. In light of the absence of any other structural difference between these two compounds, these findings suggested that the presence of the fluorine atom (at the specific site of the N-terminal biphenyl group) might exert a beneficial effect on the pharmacokinetics of inhibitor MI-1904. Fluorinated compounds are known to have a higher efficacy, higher stability, longer half-life, and better absorbability compared to their nonfluorinated counterparts, which has made the incorporation of fluorine atoms or fluorinated moieties into organic compounds a frequently used strategy during drug discovery^38–41^.

The hepatic metabolism of cynomolgus monkeys strongly resembles that of humans, both in CYP expression and in drug uptake of hepatocytes, making these animals valuable models for the prediction of human in vivo hepatic clearance^42–44^. Comparing our depletion data in human hepatocytes with previous measurements conducted on Phe(3-Am) derivatives in cynomolgus monkey hepatocytes, biotransformation of these inhibitors appeared to be more extensive in human hepatocytes. In cynomolgus monkey hepatocytes, significant conversion of the inhibitors MI-463, MI-472, MI-485 and MI-1900 was seen after 60–120-min treatments, while these same inhibitors exhibited significant levels of depletion within a mere 30 min of treatment in human hepatocytes^8^. Despite the variations in depletion rates, it remains evident that the depletion of these inhibitors in human hepatocytes closely mirrors that was observed in cynomolgus monkey hepatocytes. Interestingly, inhibitor MI-1900 was depleted at a faster rate than most other compounds in both species, while inhibitors MI-463, MI-472, and MI-485 exhibited a slower rate of depletion in cynomolgus monkey hepatocytes. On the other hand, there were less similarities in the depletion of these compounds between human and dog or rat hepatocytes, as the hepatic CYP expression and drug uptake of these species bear a weaker resemblance to those of humans^15^.

The antiviral potential of this type of protease inhibitor against IAVs or SARS-CoV-2 has been demonstrated in previous studies. In Calu-3 cells, a human respiratory epithelial cell line often used to investigate respiratory infections, compound MI-432 exhibited notable efficacy in inhibiting the proteolytic activation and replication of H1N1 and H3N2 IAV strains^45^. Furthermore, both compounds MI-432 and compound MI-1900 demonstrated a robust, dose-dependent capacity to impede the multicycle replication of SARS-CoV-2 in Calu-3 cells. At a concentration of 50 μM, they caused a significant reduction of viral titers, with a 75-fold decrease at 24 h p.i. for MI-432 and a 35 to 280-fold reduction at 24 h p.i. for MI-1900, as compared to control samples without influencing Calu-3 cell viabilitiy significantly^46^. Inhibitor MI-21, featured in the present study, has previously shown a promising inhibition of the replication of H1N1 PR8 in Calu-3 cells^47^. Additionally, a series of matriptase inhibitors was assessed against H9N2 in MDCK-II cells, a kidney epithelial cell line of canine origin. These inhibitors including MI-1900 exhibited a concentration-dependent effect on virus propagation, strongly inhibiting its replication at concentrations of 20 μM and 50 μM, with the most pronounced reduction observed for inhibitors MI-485 and MI-1904 and at the same time they did not show cell cytotoxic effect^48^.

In our study MI-463 was proven to show antiviral activity in vitro against two IAV strains. Suppression of H1N1pdm replication by MI-463 in Calu-3 cells, however, was less efficient compared to reduction in H9N2 titers in MDCK-II cells, which might be explained by variations in cell-type-dependent expression levels of matriptase and TMPRSS2 and different cellular uptake of the inhibitor in spite of the same high affinity of MI-463 towards matriptase (Ki = 0.004 µM) and TMPRSS2 (Ki = 0.003 µM). MI-1900 with low Ki values for matriptase and for TMPRSS2 (0.038 µM and 0.079 µM, respectively) was also found to have significant anti-H1N1 activity^48^.

In vitro findings underscore the potential of various matriptase inhibitors to curtail viral spread, targeting both SARS-CoV-2 and a variety of IAVs in Calu-3 cells as well as in MDCK-II cells.

Nevertheless, it is important to note that the scope of such assays remains relatively limited to date. Given the diverse array of viruses that could potentially be affected by these inhibitors, and the expanding repertoire of matriptase inhibitors available for testing, conducting comprehensive in vitro inhibition assays encompassing a spectrum of viruses, inhibitors, and cell types emerges as an imperative and promising avenue for future research. It is evident that different TTSP inhibitors, e.g., against matriptase and/or TMPRSS2, will manifest distinct effects on various viruses, making this a compelling area for further discovery.

## Conclusions

Structurally related TMPRSS2/matriptase inhibitors were tested in vitro to assess their interactions with CYP isoenzymes, depletion rates, PK parameters (elimination half-life, intrinsic clearance, hepatic extraction ratio, and bioavailability), and inhibitory effects on H1N1 and H9N2 strains of IAVs in Calu-3 and MDCK-II cell cultures. Inhibitor MI-463 emerged as an inhibitor of both H1N1 and H9N2 IAVs, showing notable inhibition of H1N1, and fully preventing the formation of H9N2 viral foci at a 50 µM concentration. In vivo PK predictions demonstrated MI-463 to be one of the compounds with greater stability, a finding reinforced by the modest depletion rate (66.7% of the parent compound retained after 120 min incubation) in primary human hepatocytes. MI-463 also possesses inhibitory effects on the CYP3A4 enzyme, suggesting increased potential for drug-drug interactions in concomitant administration with other compounds metabolized by the same pathway. Inhibitor MI-1900 also expresses notable inhibition of H1N1 and H9N2 influenza viruses and presents similar PK characteristics to inhibitor MI-463, albeit with a shorter half-life and a higher intrinsic clearance value. Both inhibitors stand out as potential drug candidates for future testing as suppressors of TMPRSS2/matriptase to prevent viral entry by certain IAVs and coronaviruses into host cells. The ability to assess PK properties, characterize the inhibitors, and perform viral inhibition assays in MDCK-II and Calu-3 cells prior to preclinical animal experiments ensures better preselection of drug candidates, since only those with suitable safety levels and PK properties are selected for further testing.

### Supplementary Information

Supplementary Figure 1.

Supplementary Figure 2.

## Data Availability

All raw data supporting the results of the present study can be achieved from the corresponding author upon reasonable request.
