# Supplementary material for: PK/PD investigation of antiviral host matriptase/TMPRSS2 inhibitors in cell models
Source: Sci Rep. 2024 Jul 18;14:16621. doi: 10.1038/s41598-024-67633-2 (PMC11258351; doi:10.1038/s41598-024-67633-2)
Supplement: Supplementary file 1 — Supplementary Figure 1. [file 41598_2024_67633_MOESM1_ESM.docx]

**Supplementary Figure S1.** Cytotoxicity of MI-463 and MI-1900 at 50 µM for 24 h in primary human hepatocytes. DMSO was applied as solvent control at 0.5% for 24 h. The data show the mean absorbance values compared to a control % ± SD in absence of inhibitor. No significant differences in cell viabilities were found between the treated and the control groups (p > 0.05).
