# Supplementary material for: PK/PD investigation of antiviral host matriptase/TMPRSS2 inhibitors in cell models
Source: Sci Rep. 2024 Jul 18;14:16621. doi: 10.1038/s41598-024-67633-2 (PMC11258351; doi:10.1038/s41598-024-67633-2)

**Supplementary Figure S2.** Predicted binding modes of compounds MI-463, MI-472, MI-1903 and MI-1904 in the active site and the peripheral pocket.


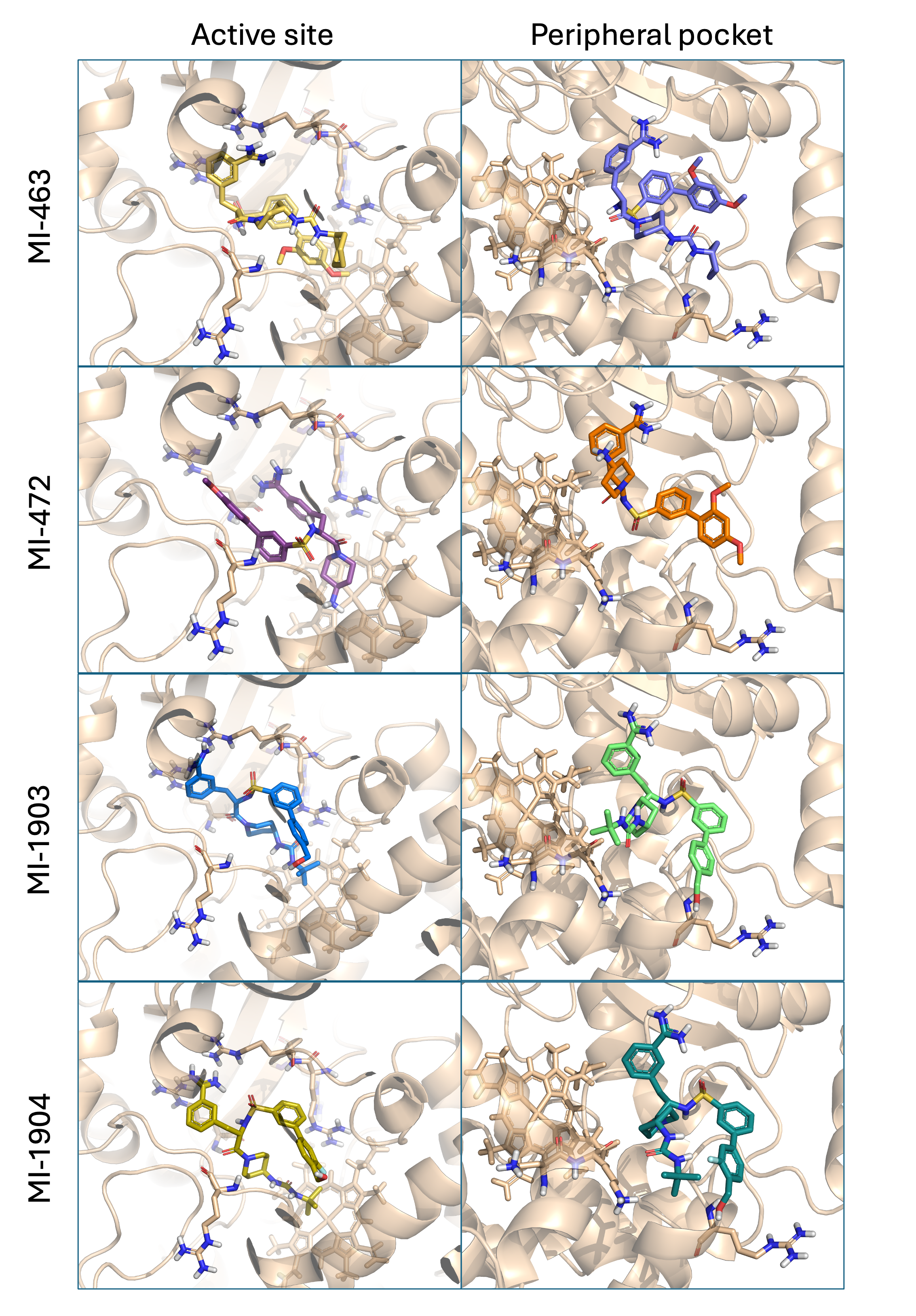

Supplement: Supplementary file 2 — Supplementary Figure 2. [file 41598_2024_67633_MOESM2_ESM.docx]
